# CSF phospho-tau levels at Parkinson’s disease onset predict the risk for development of motor complications

**DOI:** 10.1007/s00415-025-13325-4

**Published:** 2025-08-13

**Authors:** Jacopo Bissacco, Giulia Di Lazzaro, Roberta Bovenzi, Giulia Maria Sancesario, Matteo Conti, Clara Simonetta, Davide Mascioli, Maria Mancini, Veronica Buttarazzi, Mariangela Pierantozzi, Massimo Pieri, Sergio Bernardini, Alessandro Stefani, Anna Rita Bentivoglio, Paolo Calabresi, Nicola Biagio Mercuri, Tommaso Schirinzi

**Affiliations:** 1 Department of Systems Medicine, University of Rome “Tor Vergata”, Via Montpellier, 00133 Rome, Italy; 2 Institute of Neurology, Fondazione Policlinico Universitario A. Gemelli IRCCS, Rome, Italy; 3 Clinical Neurochemistry Unit and Biobank, IRCCS Fondazione Santa Lucia, European Centre for Brain Research, Rome, Italy; 4 Division of Clinical Biochemistry and Clinical Molecular Biology, University of Rome Tor Vergata, Rome, Italy; 5 UOSD Parkinson Centre, Tor Vergata University Hospital, Rome, Italy; 6 Department of Neuroscience, Università Cattolica del Sacro Cuore, Rome, Italy

**Keywords:** Parkinson’s disease, Cerebrospinal fluid, Motor complications, Tau, Nigrostriatal degeneration

## Abstract

**Background:**

Motor complications (MC), including fluctuations, represent a disabling milestone of Parkinson’s disease (PD) course, although the underlying early pathophysiological mechanisms remain unclear. We therefore investigated whether the biological profile at PD onset, as defined through a panel of CSF biomarkers, may predispose to the development of MC.

**Methods:**

We conducted a dual-center retrospective longitudinal study involving 131 de novo (DN) PD patients (newly diagnosed, untreated). At baseline, patients were evaluated by motor and non-motor scores, and the measurement of CSF total α-synuclein (α-syn), total and phosphorylated-181-tau (t-tau, p-tau), amyloid-β42 and amyloid-β40 (Aβ42, Aβ40) levels, p-tau/t-tau, Aβ42/Aβ40, and p-tau/Aβ42 ratios. According to the successive development of MC, patients were classified as “with MC” (wMC) or “without MC” (noMC). A control group of 107 controls was also collected. Variables were compared between groups, adjusting for main covariates; ROC and Cox analyses evaluated predictive values.

**Results:**

The DN PD cohort was followed for 57 (± 18) months, with 38 (29%) patients developing MC. At baseline, DN patients showed lower CSF total α-syn and t-tau levels than controls. The wMC group had higher p-tau, p-tau/t-tau, and p-tau/Aβ42 ratios than noMC. The p-tau/t-tau ratio best predicted MC development; above the cutoff of 0.148, MC were 2.6 times more likely with 81% sensitivity and 61% specificity (AUC = 0.79).

**Conclusions:**

Elevated CSF p-tau/t-tau ratio in DN PD patients predicts higher MC risk, supporting biomarker-based stratification for patients at onset. Our findings also highlight Alzheimer’s co-pathology, especially tauopathy, as a key factor in shaping PD motor progression from early stages.

**Supplementary Information:**

The online version contains supplementary material available at 10.1007/s00415-025-13325-4.

## Background

Parkinson’s disease (PD) is a neurodegenerative disorder whose neuropathological hallmarks are the accumulation of α-synuclein (α-syn) enriched Lewy bodies and the loss of dopaminergic neurons in the *substantia nigra pars compacta* (SNc) [1]. However, PD is a complex disorder in which the variegate clinical presentations reflect the profound heterogeneity of the pathogenic mechanisms and neurodegeneration patterns. Indeed, the PD syndrome encompasses both motor and non-motor disturbances [2], the first basically arising from the impairment of the dopaminergic nigrostriatal transmission and the latter from the disruption of multiple extra-motor brain networks [3]. Accordingly, dopaminergic drugs, mostly levodopa, dramatically ameliorate motor issues, ensuring effective symptomatic control, especially at the early disease stages. Later in the clinical course, instead, patients are burdened by motor complications (MC) and the motor fluctuations, namely representing the loss of linear response to dopaminergic therapy and the development of dyskinesia or akinesia phases, which set off an advanced-complicated stage of the disease[4, 5].

Animal models, neuroimaging, and neurophysiology studies allowed identifying some pathophysiological determinants of fluctuations and MC at all, such as the aberrant corticostriatal plasticity, the hyperactivity of glutamatergic transmission, the levodopa metabolism in non-dopaminergic neurons, and the chronic pulsatile dopamine receptors stimulation [6–9]. Also, the female gender and a non-tremor-dominant phenotype are well-established risk factors for the development of MC [10]; nevertheless, the early pathological substrate of this disabling phenomenon, or eventual biological predictors, which are fundamental for targeted interventions, have not been fully elucidated yet.

Recent studies highlighted brain co-pathology’s relevance to PD’s clinical trajectories [11, 12]. Indeed, aside from Lewy bodies, other protein aggregates, especially amyloid-β and tau, which both shape the so-called Alzheimer’s disease (AD)-related co-pathology, may accumulate in the brain of PD patients, with an estimated rate of 65% and 50%, respectively[11, 13], aggravating the α-syn-related neurodegeneration [14] and accelerating the cognitive deterioration [11, 15]. Amyloid-β and tau co-pathology primarily affect the cortex, although they also involve subcortical structures [13, 16]. In particular, tau seems to contribute to early steps of nigrostriatal denervation[17, 18], basically suggesting that tau co-pathology occurs precociously, even in the core PD circuits, probably influencing the successive course of MC.

The cerebrospinal fluid (CSF) levels of neurodegeneration-related proteins mirror the brain molecular events, enabling the in vivo tracking of neuropathology [19, 20]. Accordingly, here we used the CSF biomarkers to investigate how the earliest pathological changes may predispose to the later development of fluctuations in PD patients. We thus conducted a longitudinal study on a dual-center cohort of newly diagnosed, untreated PD patients (“de novo”) with a baseline assessment of amyloid peptides, tau proteins, and α-syn CSF levels to estimate the association between the CSF-based co-pathology profile and the onset of MC along the disease course.

## Methods

### Study population

The study involved 131 PD patients and 107 control subjects recruited at the Neurology Unit of the Tor Vergata University Hospital (which recruited 104 PD patients and 107 controls) and the Neurology Unit of Fondazione Policlinico Universitario Agostino Gemelli (which recruited 27 PD patients); both in Rome, Italy, between 2015 and 2021. PD diagnosis was made by movement disorder specialists following the 2015 MDS Criteria. The control group included subjects with other non-neurodegenerative and non-inflammatory conditions receiving lumbar puncture for diagnostic purposes (e.g., headache, functional disorders, or PNS diseases). Biological diagnosis of Alzheimer’s disease (AD) was excluded according to revised criteria [21]. Subjects with main acute/chronic internal/inflammatory/infectious disease were excluded from the study to avoid potential biases. For each participant, demographic, clinical, and anthropometric information was recorded.

Patients all had a baseline assessment performed at the time of diagnosis, before starting symptomatic treatment (as de novo, DN), including a lumbar puncture for CSF biomarkers measurement and a deep clinical evaluation, and a successive regular follow-up of two visits per year for a minimum 2 years. Baseline scores encompassed the MDS Unified Parkinson’s Disease Rating Scale Part III and IV (MDS-UPDRS III—IV), the Hoehn and Yahr scale (H&Y), the Mini-Mental State Examination (MMSE) adjusted for age and educational level, the Montreal Cognitive Assessment (MoCA), and the Non-motor Symptoms Scale (NMSS). All patients also underwent 3 T brain MRI to rule out secondary causes of parkinsonism or atypical parkinsonism biomarkers.

Patients were regularly followed up through one to two visits per year and treated according to standard clinical practice. The possible appearance of signs and symptoms suggestive of atypical parkinsonisms (as defined by current diagnostic criteria for progressive supranuclear palsy, corticobasal degeneration, multisystem atrophy, Lewy bodies dementia) was excluded by a complete neurological examination at each visit. According to the development of MC over the follow-up (FU) period, defined by a stable transition (lasting more than 3 months) of the MDS-UPDRS IV (items 4.1, 4.2, 4.3, 4.4, 4.5) from 0 to ≥ 1 (data available for all patients), PD patients were divided into two groups, one with MC (wMC) and one without MC (noMC). The levodopa equivalent daily dose (LEDD, mg/day) [22], was recorded at the MC onset for wMC patients and at the last visit for noMC patients.

### Biomarkers assay

CSF was withdrawn within one week from clinical assessment by lumbar puncture following standard procedures [23, 24]. The levels of amyloid-β42 (Aβ42), amyloid-β40 (Aβ40), total tau (t-tau), and phosphorylated-181-tau (p-tau) were measured using fully-automized CLEIA Fujirebio LUMIPULSE® G1200 (Fujirebio, Inc., Tokyo, Japan), and the levels of total α-syn were assessed using Human α-synuclein ELISA kit (Biolegend). Because of the biological significance, the following biomarker ratios were also calculated for each group: Aβ42/Aβ40, p-tau/t-tau, p-tau/Aβ42.

### Statistical analysis

Continuous variable distribution was preliminarily assessed by the Shapiro–Wilk test. The non-normally distributed ones were IDF.NORMAL (fractional rank, mean, standard deviation)-transformed when needed (details in the reference [25]), and then further reassessed for normality to allow for statistical calculation. Categorical variables were compared between groups using the Chi-square test. Continuous clinical and biological variables were compared between groups using one-way analysis of variance (ANOVA) or a one-way analysis of covariance (ANCOVA) adjusted for potential confounding factors. Prognostic accuracy was initially estimated by receiver operating characteristic (ROC) curve analysis, computing the area under the curve (AUC) along with the corresponding 95% confidence interval (CI), which was derived using a Bootstrap method (1000 resamples). A corrected model including sex, age, and disease duration was then used to evaluate adjusted diagnostic performance. The optimal sensitivity and specificity were calculated based on the Youden Index. The computed Youden Index was converted to raw data and then used to dichotomize our sample to compute hazard ratios (HR) through univariate Cox Regression, and to receive Kaplan–Meier curves depending on the respective cutoff value. Kaplan–Meier curves were compared with log-rank tests.

Statistical analysis was run blind by using IBM-SPSS-29(Armonk, NY, USA) and with R-Studio (packages: pROC, survival). Significance was set at *p* < 0.05.

## Results

### Clinical features and outcomes

Table 1 summarizes the baseline clinical-demographic parameters of the study population. These features did not differ between the two enrolling centers. All 131 DN patients were longitudinally followed up for a mean ± st.dev of 57 ± 18 months (24 months minimum). The 29% (38 patients) developed MC (wMC) at a mean time of 51.38 ± 15.77 months from the diagnosis, while the 71% (93 patients) did not develop MC (noMC). The baseline clinical scores did not differ between wMC and noMC patients; as well, the FU time was similar between the groups, while the LEDD at MC onset and at last visit for wMC and noMC patients, respectively, was higher in wMC than in noMC (t(115) = 4.90, *p* < 0.001), even in the model adjusted for age, sex, and disease duration (F(1, 115) = 24.23, *p* < 0.001, ηp2 = 0.179) (Table 2**).**

**Table 1:** Demographic and clinical characteristics of PD patients

	DN (*n* = 131)	CTR (*n* = 107)	Significance
Demographic parameters			
Sex (m/f)	94/37	61/46	n.s
Age (y)	61.65 ± 10.332 (32–81)	63.50 ± 11.05 (25–79)	n.s
FU (mo)	57.99 ± 19.23 (22–127)	\	\
Clinical parameters			
Disease Duration (y)*	1.510 ± 1.0254 (0–7.0)	\	**\**
LEDD (mg/day)	0	\	**\**
MDS-UPDRS part-III	23.48 ± 11.56 (5.00–60.00)	\	**\**
HY	1.90 ± 0.59 (1.00–4.00)	\	**\**
MMSE	27.98 ± 2.11 (19.00–30.00)	\	\
MoCA	25.16 ± 3.66 (12.00–30.00)	\	\
NMSS *total score*	37.40 ± 30.69 (0.00–140.00)	\	**\**
CSF biomarkers			
α-Synuclein (pg/ml)^a^	926.6 ± 318.7 (238–1646)	1201.22 ± 498.599 (666–2669)	***p* = 0.022**
Aβ42 (pg/ml)	895.85 ± 343.34 (195–1807)	938.02 ± 493.932 (26–2640)	n.s
Aβ40 (pg/ml)^b^	7500.11 ± 3010.42 (1497–16,532)	7623.56 ± 3321.676 (1871–16,127)	n.s
Aβ42/Aβ40^b^	0.13 ± 0.06 (0.04–0.50)	0.15 ± 0.05 (0.04–0.30)	n.s
Total tau (pg/ml)	211.86 ± 104.90 (50–806)	274.23 ± 186.23 (57–1052)	***p* = 0.024**
Phosphorylated-181-tau (pg/ml)	31.51 ± 16.43 (2–124)	36.29 ± 20.207 (6–150)	n.s
Phosphorylated-181-tau/total tau	0.16 ± 0.07 (0.02–0.40)	0.16 ± 0.07 (0.03–0.39)	n.s
Phosphorylated-181-tau/Aβ42	0.04 ± 0.03 (0.00–0.21)	0.15 ± 0.05 (0.01–0.95)	n.s

Values are given in mean ± standard deviation (range)

Statistical significance is marked in bold

*n* number, *y* years, *m* male, *f* female, *DN* de novo PD patients, *FU* follow-up, *LEDD* Levodopa-equivalent daily dose, *MDS-UPDRS III* Movement disorders society-sponsored revision of the unified Parkinson’s disease rating scale part III, *HY* Hoehn & Yahr, *MMSE* MiniMental state examination, *MoCA* Montreal cognitive assessment, *NMSS* Non-motor symptoms scale

^*^From motor symptoms onset to diagnosis

^a^Data are available for 58 PD patients and 27 HC

^b^Data are available for 102 PD patients and 59 HC

**Table 2:** Demographic and clinical characteristics of wMC and noMC cohorts

	wMC (*n* = 38)	noMC (*n* = 93)	significance
Demographic parameters			
Sex (m/f)	27/11	67/26	n.s
Age (y)	60.65 ± 8.703 (41–73)	62.04 ± 10.932 (32–81)	n.s
FU (mo)	51.38 ± 15.77 (22–85)	61.01 ± 19.99 (27–127)	n.s
Clinical parameters			
Disease Duration (y)*	1.51 ± 0.98 (0–5.0)	1.51 ± 1.05 (0–7.0)	n.s
Time to MC onset (mo)	48.67 ± 14.53 (22–79)	\	\
LEDD at MC onset/end FU (mg/day)	667.98 ± 291.83 (150–1300)	416.173 ± 173.04 (150–925)	***p* < 0.001**
MDS-UPDRS part-III	22.52 ± 9.62 (5–46)	23.37 ± 11.25 (5–47)	n.s
HY	1.93 ± 0.56 (1.0–3.0)	1.93 ± 0.56 (1.0–3.0)	n.s
MMSE	28.06 ± 1.80 (25.00–30.00)	27.95 ± 2.30 (19.00–30.00)	n.s
MoCA	23.30 ± 5.35 (12–30)	25.50 ± 3.22 (12–30)	n.s
NMSS *total score*	43.50 ± 37.50 (0–140)	34.55 ± 27.31 (0–123)	n.s
CSF biomarkers			
α-Synuclein (pg/ml)^a^	952.85 ± 307.07 (475–1620)	914.73 ± 326.91 (238–1646)	n.s
Aβ42 (pg/ml)	816.43 ± 308.18 (285–1729)	928.30 ± 353.13 (195–1807)	n.s
Aβ40 (pg/ml)^b^	7882.34 ± 2469.51 (1497–11,833)	7395.00 ± 3148.55 (2853–16,532)	n.s
Aβ42/Aβ40^b^	0.12 ± 0.092 (0.04–50)	0.1310 ± 0.046 (0.05–23)	***p* = 0.014**
Total tau (pg/ml)	202.22 ± 130.90 (60–806)	206.27 ± 75.85 (39.00–377)	n.s
Phosphorylated-181-tau (pg/ml)	39.59 ± 20.78 (8–124)	27.75 ± 12.44 (5.00–59.00)	***p* < 0.001**
Phosphorylated-181-tau/total tau	0.22 ± 0.087 (0.11–0.40)	0.1371 ± 0.048 (0.02–0.26)	***p* < 0.001**
Phosphorylated-181-tau/Aβ42	0.056 ± 0.039 (0.02–0.19)	0.033 ± 0.023 (0.00–0.21)	***p* < 0.001**

Values are given in mean ± standard deviation (range)

Statistical significance is marked in bold

^*^From motor symptoms onset to diagnosis

^a^Data are available for 18 wMC patients and 40 NoMC

^b^Data are available for 22 wMC patients and 80 NoMC

### Biochemical differences between groups

At baseline, DN patients had lower CSF levels of α-syn (F(1, 82) = 5.42, p = 0.022) and t-tau (F(1, 229) = 5.18, *p* = 0.024) than controls, even in the model adjusted for age and sex (F(1, 78) = 4.163, *p* = 0.045, ηp2 = 0.051, F(1, 230) = 3.988, *p* = 0.047, ηp2 = 0.017 respectively (Supplemental Fig. 1). No differences resulted in other CSF biomarkers.

**Fig. 1:**
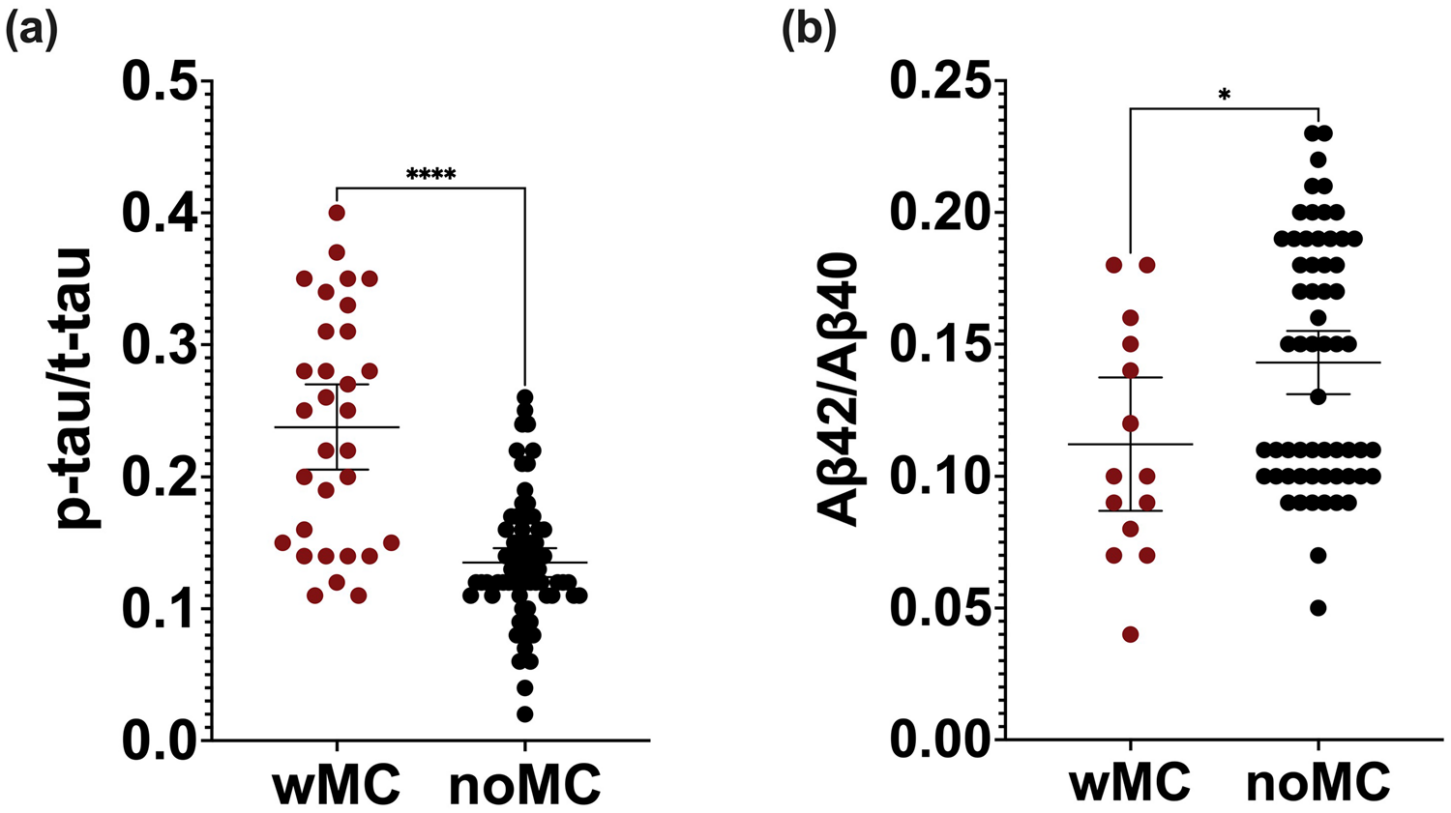
Scatter plots of CSF phosphorylated-181 tau/total tau (p-tau/t-tau) and amyloid-β42/amyloid-β40 (Aβ42/Aβ40) values in patients with motor complications (wMC) and without motor complications (noMC), with median and 25th-75th interquartile range. *p < 0.05. **a** CSF p-tau/t-tau values comparison. Black dots represent noMC cohort; red dots represent wMC cohort. **b** CSF Aβ42/Aβ40 values. Black dots represent noMC cohort; red dots represent wMC cohort

Stratifying DN patients according to the successive development of MC, we found that, at baseline, wMC patients had higher CSF p-tau levels (F(1, 128) = 15.13, *p* < 0.001), p-tau/Aβ42 (F(1, 128) = 23.93, *p* < 0.001), and p-tau/t-tau (F(1, 128) = 48.42, *p* < 0.001) ratios than noMC ones, even in the adjusted model (F(1, 100) = 19.61, *p* < 0.001, ηp2 = 0.164; F(1, 101) = 27.94, *p* < 0.001, ηp2 = 0.217; F(1, 100) = 31.71, *p* < 0.001, ηp2 = 0.241; respectively). wMC patients also had lower CSF Aβ42/Aβ40 ratio (F(1, 99) = 6.21, *p* = 0.014) compared to noMC, although the result was lost in the adjusted model (Fig. 1).

### Receiver operating characteristics (ROC) analysis

**Fig. 2:**
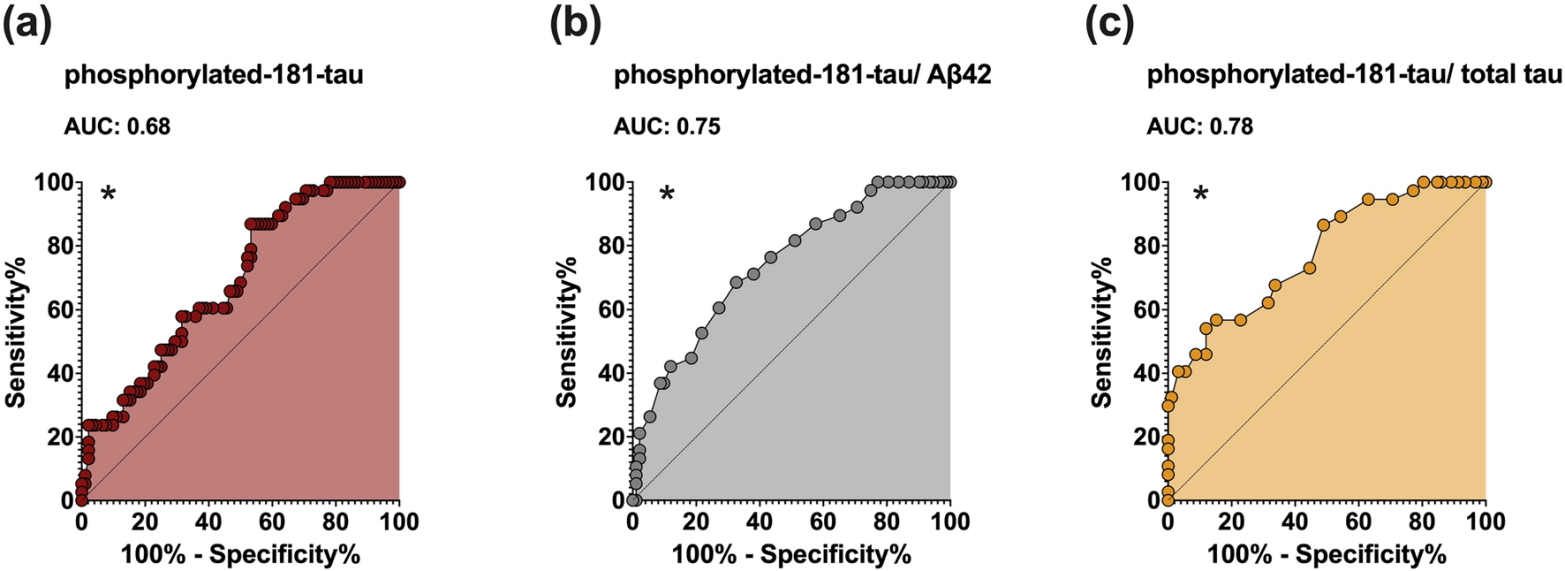
Receiver operating characteristic (ROC) curves of CSF phosphorylated-181 tau levels (p-tau), phosphorylated-181 tau/amyloid-β42 (p-tau/Aβ42), and phosphorylated-181 tau/total tau (p-tau/t-tau). *p < 0.05. **a** ROC curve of cerebrospinal fluid p-tau levels to discriminate wMC and noMC groups (red). **b** ROC curve of cerebrospinal fluid p-tau/Aβ42 values to discriminate wMC and noMC groups (gray). **c** ROC curve of cerebrospinal fluid p-tau/t-tau values to discriminate wMC and noMC groups (yellow)

To estimate the association between the CSF biomarker profile at PD onset (in DN patients) and the risk of developing MC along the disease course, we fitted ROC curves and calculated cutoff values discriminating between patients wMC and noMC. The areas under the curve (AUC) for CSF p-tau, p-tau/Aβ42, and p-tau/t-tau were 0.68 (CI 95%: 0.58–0.78), 0.75 (CI 95%: 0.66–0.84), and 0.78 (CI 95%: 0.69–0.87), respectively. Given the highest AUC value for the p-tau/t-tau ratio, we implemented the analysis considering sex, age, and disease duration as covariates, obtaining an AUC of 0.79 (0.71–0.88); the optimal cutoff value was 0.148 (derived from a Z-score of 0.153), resulting in a sensitivity of 81% and a specificity of 61% (Fig. 2). The *Supplementary Information* file contains the calculation of Positive Predictive Value (PPV), Negative Predictive Value (NPV), and accuracy of the identified cut-off value in predicting MC.

### Cox regression for hazard ratio (HR) and survival curves

**Fig. 3:**
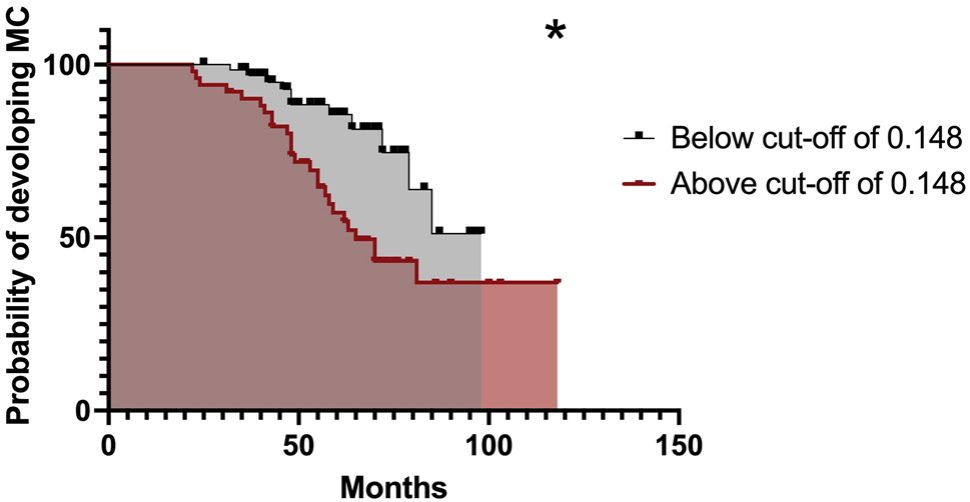
Survival curve for the onset of motor complications during follow-up. The groups for the Kaplan–Meier estimates were determined by maximizing the Youden index for adjusted p-tau/t-tau values. *p < 0.05. The cutoff value shows a highly significant difference in motor complication onset for the resulting groups (*p* = 0.007). Groups below the cutoff are depicted in gray and above the cutoff in red

Stratifying DN patients according to CSF p-tau/t-tau ratio cutoff value, we found that the risk of developing MC was 2.6 times higher (χ2(1) = 7.36, *p* = 0.007) in patients with p-tau/t-tau ratio above 0.148. The main event (occurrence of MC) differed significantly between patients above and below the cutoff value (*p* = 0.006 Log-rank test). DN patients with a p-tau/t-tau ratio ≥ 0.148 developed MC after a mean time of 69 months from the onset, whereas patients under the cutoff developed MC 86 months later (Fig. 3).

## Discussion

This study aimed to estimate the associations between the CSF-based AD-related co-pathology profile at PD onset and the risk for MC development along the PD progression. Of interest, we found that DN patients with more pronounced tau and amyloid-β pathology, as shaped by the higher CSF p-tau, p-tau/t-tau, and p-tau/Aβ42 ratios, and the lower Aβ42/Aβ40 ratio, may develop more frequently and earlier MC in the disease course.

AD-related co-pathology is an emerging theme in PD and Lewy bodies-associated diseases since several lines of evidence have now demonstrated that pathological proteins act synergistically, contributing to an accelerated disease trajectory. Overt AD-related co-pathology is often observed in the brains of patients at late PD stages or Lewy body dementia. However, the molecular interactions might begin earlier, affecting the synucleinopathy spreading and the neurodegeneration burden since the earliest stages of the clinical-pathological course. [14, 18]

Tau seems to have an intimate and precocious relationship with α-syn, as the two proteins cooperate in triggering synaptopathy, reciprocally facilitate the aggregation into fibrillary structures, and colocalize within the Lewy bodies [14, 18]. According to the hypothesis of coprecipitation in pathological aggregates, in our cohort, the DN patients’ CSF levels of both t-tau and α-syn were lower than controls, aligning with other previous findings [26, 27]. The CSF levels of tau species, total and phosphorylated, may also reflect biological processes other than the sequestration in aggregates, including the extracellular release following neuronal loss and age-related or pathology-specific phenomena [19, 23]. In fact, no univocal and straightforward associations exist between CSF t-tau levels and clinical manifestations in PD [20]. However, in single studies, the higher CSF tau levels were correlated with greater clinical severity, especially in cognitive domains [28], suggesting that tau pathology may contribute to the pathophysiology of PD phenomenology, although the CSF levels do not allow for a linear readout of such complex events.

Here, p-tau levels, p-tau/t-tau, and p-tau/Aβ42 ratios were higher in patients who went on to develop MC (wMC patients) than in those who did not develop MC (noMC). The p-tau levels, more than the t-tau, specifically reflect tau neurofibrillary tangles accumulation. In contrast, the p-tau/Aβ42 ratio, which combines two different pathological processes into a single measure (one related to neurofibrillary tangles and one to amyloid plaques), has good accuracy in tracing the core AD-related co-pathology overall[29, 30]. The p-tau/t-tau ratio, as well, allows, to some extent, for discriminating AD-related tauopathy from non-AD tauopathy [31, 32]. Therefore, DN patients who developed MC had a significant increase in all the tau pathology indexes that support an involvement of tau even in motor progression of PD, as observed in other works [27].

In particular, we noticed that the accuracy of tauopathy markers in identifying wMC patients was milder for p-tau alone (AUC = 0.68) and higher for the p-tau/Aβ42 ratio (AUC = 0.75) and p-tau/t-tau ratio (AUC = 0.78), the latter being the best predictor for the development of MC in DN patients in the first 5 years (2.6 higher risk in patients above the cut-off value of 0.148). These findings thus reveal how a likely incipient AD-related tau pathology may occur in the motor network of PD patients since the earliest disease stages, at onset indeed, accounting for a greater risk of MC.

The nigrostriatal denervation is a major determinant in MC pathophysiology, and the synucleinopathy, although critical for the progression of neurodegeneration within this pathway, seems not directly to impact their development and maintenance [9]. Actually, also in our cohort, CSF α-syn levels did not differ between wMC and noMC patients, matching results from other independent studies [9]. On the other hand, there is proof that pathological tau accumulates in the nigrostriatal route since the very early disease stages, taking part in its degeneration and contributing to motor disturbances [18]. Accordingly, tau pathology, rather than synucleinopathy, might be critical in leading the development of MC. However, because the CSF biomarkers prevent a topographical definition of the biological processes, we cannot establish at which level of the motor network the tauopathy intervenes since it may also localize in the cortex [16], disrupting cortical projections instead of the subcortical pathways.

Amyloid-β pathology, conversely, had lesser weight in these dynamics. The wMC patients exhibited a CSF Aβ42/Aβ40 ratio lower than noMC, although the significance was lost when adjusting for the covariates. Low Aβ42 levels or Aβ42/Aβ40 ratio are known to reliably predict cognitive decline and non-motor symptoms at all in PD patients, even from the early phases [33, 34]. Of interest, they also identify PD patients with pronounced axial motor disturbances [35, 36], supporting how the AD-related co-pathology might impact the motor phenotype of PD, basically aggravating the long-term motor complications.

This study has limitations due to the sample size and the low number of patients developing MC during a relatively short observation time. Moreover, grouping dyskinesias and other motor fluctuations under the broad umbrella of ‘motor complications’ may be overly simplistic and potentially introduce bias. Another limitation is that the control group included not healthy subjects but patients with other non-neurodegenerative and non-inflammatory neurological conditions. We should also acknowledge the raw assessment for the detection and evaluation of MC or the absence of longitudinal complete motor and cognitive scores that might have allowed for a broader characterization of the cohort’s clinical progression. In addition, the full CSF biomarker panel was available only for a subgroup of PD patients and controls, did not include the phosphorylated-tau-217, which is a very sensitive index of AD-related tau pathology[37, 38], or a quantitative α-syn seeding amplification assay (SAA), which would be critical for the sensitive in vivo quantification of Lewy body pathology [39]. Conversely, we could operate on a very homogeneous and selected DN cohort, with a dual-center enrollment and regular follow-up, which provided enough reliability for our findings.

We demonstrated that AD-related co-pathology CSF biomarkers might be useful in stratifying the risk for MC development in DN PD patients. In particular, the p-tau and the p-tau/t-tau ratio exhibited the highest value, although the statistical strength prevents straightforward conclusions. While being promising for clinical practice, from the perspective of tailored treatments, these findings also suggest the relevance of AD-related co-pathology, especially tauopathy, to the motor progression and complications of PD, showing how the molecular background and the biological profile may trace the long-term path since the disease onset. Now, further confirmatory studies with appropriate longitudinal design, clear-cut fluctuations classification, replication cohorts, and additional biomarkers are needed.

## Supplementary Information

Below is the link to the electronic supplementary material.

Supplementary file1 (DOCX 156 KB)

## Data Availability

The datasets used and analyzed during the current study are available from the corresponding author upon reasonable request.
