# Supplementary material for: CSF phospho-tau levels at Parkinson’s disease onset predict the risk for development of motor complications
Source: J Neurol. 2025 Aug 13;272(9):573. doi: 10.1007/s00415-025-13325-4 (PMC12350592; doi:10.1007/s00415-025-13325-4)
Supplement: Supplementary file 1 — Supplementary file1 (DOCX 156 KB) [file 415_2025_13325_MOESM1_ESM.docx]

**Supplementary information**

*Abbreviation List (alphabetically listed)*

- *α-syn - α-synuclein;*
- *CTR - Controls;*
- *MC - Motor Complications;*
- *noMC - patients without Motor Complications;*
- *NPV - Negative Predictive Value;*
- *p-tau - Phosphorylated-181-tau;*
- *PD - Parkinson’s Disease;*
- *PPV - Positive Predictive Value;*
- *t-tau - Total-tau;*
- *wMC - patients with Motor Complications.*

**1. Group analysis between PD and CTR**

**Supplemental Fig.1** Scatter plots of CSF total α-synuclein (total α-syn) and total-tau (t-tau) values in patients with Parkinson’s disease (PD) and controls (CTR), with median and 25^th^-75^th^ interquartile range. *p < 0.05. (a) CSF total α-syn values comparison. Black dots represent CTR cohort; red dots represent PD cohort. (b) CSF t-tau values. Black dots represent CTR cohort; red dots represent PD cohort.


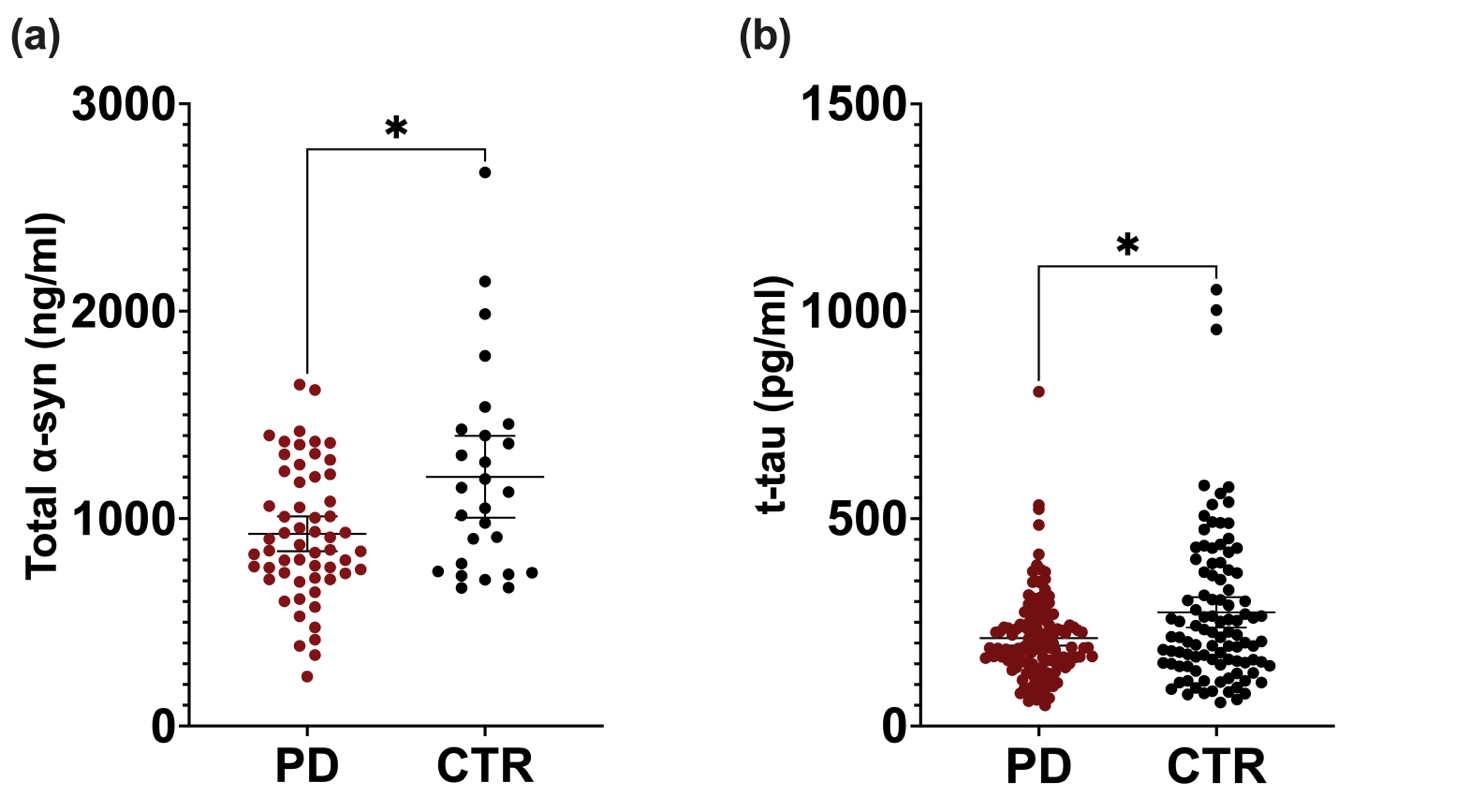


**2. Calculation of PPV**

**Supplemental Table1**. presents the distribution of patients according to the p-tau/t-tau ratio threshold of 0.148, stratified by the presence (wMC) or absence (noMC) of MC. This cut-off was derived from the ROC curve of the p-tau/t-tau ratio using the Youden index.

|  | wMC patients | noMC patients |
| --- | --- | --- |
| p-tau/t-tau ratio < 0.148 | 12 | 62 |
| p-tau/t-tau ratio ≥ 0.148 | 26 | 31 |

PPV = 45.6%

NPV = 83.7%

Accuracy = 67.1%
